# Novel 6- and 7-Substituted Coumarins with Inhibitory Action against Lipoxygenase and Tumor-Associated Carbonic Anhydrase IX

**DOI:** 10.3390/molecules23010153

**Published:** 2018-01-12

**Authors:** Aikaterini Peperidou, Silvia Bua, Murat Bozdag, Dimitra Hadjipavlou-Litina, Claudiu T. Supuran

**Affiliations:** 1 Dipartimento Neurofarba, Sezione di Scienze Farmaceutiche e Nutraceutiche, Università degli Studi di Firenze, Via Ugo Schiff 6, 50019 Sesto Fiorentino (Firenze), Italy; peperidou@pharm.auth.gr (A.P.); silvia.bua@unifi.it (S.B.); 2 Department of Pharmaceutical Chemistry, School of Pharmacy, Faculty of Health Sciences, 54124 Thessaloniki, Greece; hadjipav@pharm.auth.gr; 3 Dipartimento di Chimica, Laboratorio di Chimica Bioinorganica, Università degli Studi di Firenze, Via della Lastruccia 3, 50019 Sesto Fiorentino (Firenze), Italy; bozdag.murat@unifi.it

**Keywords:** coumarins, carboxamides, carbonic anhydrase, lipoxygenase, enzyme inhibitor

## Abstract

A series of carboxamide derivatives of 6- and 7-substituted coumarins have been prepared by an original procedure starting from the corresponding 6- or 7-hydroxycoumarins which were alkylated with ethyl iodoacetate, and the obtained ester was converted to the corresponding carboxylic acids which were thereafter reacted with a series of aromatic/aliphatic/heterocyclic amines leading to the desired amides. The new derivatives were investigated as inhibitors of two enzymes, human carbonic anhydrases (hCAs) and soy bean lipoxygenase (LOX). Compounds **4a** and **4b** were potent LOX inhibitors, whereas many effective hCA IX inhibitors (K_I_s in the range of 30.2–30.5 nM) were detected in this study. Two compounds, **4b** and **5b**, showed the phenomenon of dual inhibition. Furthermore, these coumarins did not significantly inhibit the widespread cytosolic isoforms hCA I and II, whereas they were weak hCA IV inhibitors, making them hCA IX-selective inhibitors. As hCA IX and LOX are validated antitumor targets, these results are promising for the investigation of novel drug targets involved in tumorigenesis.

## 1. Introduction

Vertebrates, including humans, encode for a multitude of metalloenzymes belonging to the carbonic anhydrase (CA, EC 4.2.1.1) family of proteins [1,2,3,4]. Although seven CA genetic families are known to date (α-, β-, γ-, δ-, ζ-, η- and θ-CAs) [2,5], only α-CAs are present in humans, but as 15 different isoforms, 12 of which are catalytically active and involved in a multitude of physiologic functions [3,4,5,6,7,8,9]. By catalyzing the reversible hydration of CO_2_ to bicarbonate, with the release of a hydronium ion, in humans CAs are involved in pH regulation, biosynthetic reactions, electrolyte secretion, excretion, tumorigenesis, etc. [3,4,6,7,8,9]. CA inhibitors (CAIs) are in pharmacological/clinical use for decades for the treatment of glaucoma [6,7], for the imaging and treatment of hypoxic tumors [3,4,8,9], as anti-obesity agents [10], or as diuretics [11]. Recently these pharmacological agents were validated for the management of neuropathic pain [12], but the sulfonamides, which are the main class of CAIs [11,12,13] possess a rather large number of side effects, as they indiscriminately inhibit all catalytically active CA isoforms, and not only the ones targeted for a specific application [1,2,3,13,14,15,16,17]. Thus, alternative classes of CAIs to the sulfonamides and their isosteres were explored in the last period [14], which led to the discovery of several totally different inhibition mechanisms and families of inhibitors [14]. Among them, the coumarins are among the most relevant ones for several reasons [18]. Discovered initially in a natural product library isolated from an Australian biota [18], the coumarins were demonstrated to possess a very particular inhibition mechanism [18,19]. Indeed, they act as prodrug, suicide inhibitors which undergo a hydrolytic process within the enzyme active site with generation of 2-hydroxycinnamic acid derivatives [18,19]. These relatively bulky compounds cannot bind to the catalytic metal ion, which is a Zn(II) ion in α-CAs, and is situated deep within the active site [1,2,3]. Instead, the hydrolyzed coumarins were observed (by means of X-ray crystallography) to be bound at the entrance of the active site cavity, which is rather large for the hCAs [1,2,3,18,19]. Furthermore, that is the only region of the active site which is the most variable between the 12 catalytically active isoforms, which may explain why the coumarins and their derivatives are among the most isoform-selective CAIs known to date [19,20,21,22,23,24,25]. Indeed, extensive drug design campaigns in which various parts of the coumarin moiety were changed, showed the useful as well as the detrimental substitution patterns as well as the tolerated or less tolerated substituents that can be appended to the ring system in order to obtain effective and isoform-selective CAIs [18,19,20,21,22,23,24,25]. Among the most effective coumarin CAIs detected in this way it has been observed that 6- and 7- or 6,7-disubstituted derivatives possess an effective inhibition of the tumor-associated isoforms CA IX and XII, whereas they are poor inhibitors or do not significantly inhibit the widespread “house-keeping” isoforms hCA I and II (the inhibition of which is responsible for the side effects of the sulfonamide CAIs [1,2,3,4]). Thus, here we continue our research in developing non-sulfonamide CAIs and report a new series of coumarins possessing 6- and 7 moieties which have not been explored earlier, of the ether-carboxamide type. 

Lipoxygenase (LOX) plays a major role in many inflammatory diseases including chronic obstructive pulmonary disease (COPD), asthma, chronic bronchitis, cancer including pancreatic, gastric and brain tumors. Similarly to different isozymes of CA, such as CA II, LOX is expressed in pancreatic, gastric as well as brain tumors [26]. It should to be mentioned that morphological cells changes and CA activity are used to determine the effect of LOX inhibitors on cancer cell differentiation [26]. LOX is upregulated in cancer cells and arachidonic acid as well as its metabolites, 5-HETE and 12-HETE, stimulate mitogenesis of human pancreatic cancer cells. Furthermore, blockade of LOX pathways abolishes cancer cell proliferation in vitro and induces cancer cell apoptosis [27]. The development of coumarins as antioxidant agents, anticancer and LOX inhibitors has attracted much attention recently [27,28,29]. Several reviews and research papers have updated and expanded the knowledge in this field [27,28,29]. In recent years, intensive research has been conducted on creating new polyfunctional drugs [30,31]. For the treatment of complex diseases e.g., neurological disorders, cancer and inflammation, in which more than one target is implicated, a combination of drugs is frequently used. Therefore, novel potent inhibitors of both LOX and CA II are required to explore the role of these enzymes further and to enable the drug discovery efforts. Thus, we considered of interest to prepare and test new compounds as dual CA and LOX inhibitors.

## 2. Results and Discussion

A large number of variously substituted coumarins were reported to act as CAIs [18,19,20,21,22,23,24,25] and to also possess diverse other biological/pharmacological actions [30]. For example, the development of coumarins as antioxidant agents, anticancer and LOX inhibitors has attracted much attention [27,29,31,32,33]. It has been also reported that antioxidant polyphenols structurally related to coumarins effectively inhibited CAs [34]. In recent years, intensive research has been conducted for creating new polyfunctional drugs for the treatment of complex diseases, in which more than one target is implicated [30,31,32,33,34,35]. 

**Scheme 1:**
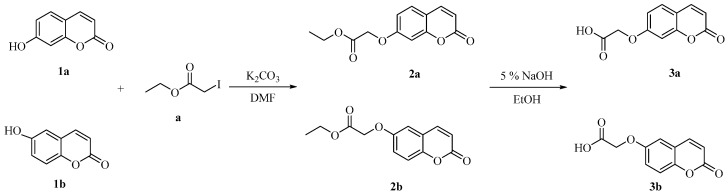
Preparation of the key intermediate carboxylic acids **3a** and **3b**.

No carboxamide derivatives of coumarins at the 6- or 7-position of the ring were explored so far. Thus, the drug design strategy was to obtain the carboxymethyl-oxy derivatives **3a** and **3b**, which possess a reactive COOH moiety, easy to derivatize with aromatic, aliphatic or heterocyclic amines, in order to generate chemical diversity. Thus, commercially available 6- or 7-hydroxy-coumarins **1a**–**b** were reacted with ethyl iodoacetate leading to esters **2a** and **2b**, which were then hydrolysed in alkaline medium to the corresponding acids **3a** and **3b** (Scheme 1). 

**Scheme 2:**
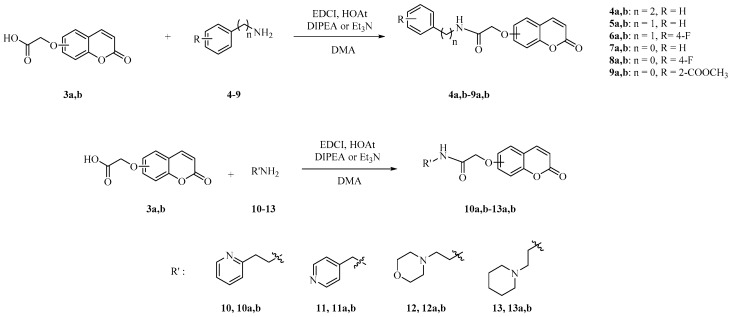
Preparation of amides **4a**,**b**–**13a**,**b** reported in the paper.

The two carboxylic acids **3a** and **3b** were converted to the corresponding amides by reaction with aromatic, aliphatic and heterocyclic primary amines, as shown in Scheme 2, by using carbodiimide chemistry. The nature of the various amines was chosen in such a way as to generate the widest possible chemical diversity (Scheme 2). All compounds were extensively characterized by spectral and other physico-chemical procedures which proved their structure (see Experimental part for details).

The new coumarins (**5a**,**b**–**6a**,**b**, **9a**,**b**–**13a**,**b**) and previously reported coumarins (**2a**–**b**, **3a**–**b**, **7a**–**b**, **8a**–**b**) were investigated here for the inhibition of four physiologically relevant CA isoforms, hCA I and II (cytosolic, widespread isoforms, involved in glaucoma and other eye diseases [1,3,6], hCA IV (membrane-bound isoform highly abundant in the kidney and lungs and involved in diuresis, respiration and retinitis [11] as well as hCA IX (tumor-associated, transmembrane isoforme, a newly validated antitumor target [3,4,9]. A stopped-flow CO_2_ hydrase assay has been used for monitoring the inhibition of these CAs with the new coumarins and acetazolamide (**AAZ**, 5-acetamido-1,3,4-thiadiazole-2-sulfonamide, a clinically used CAI) as standard inhibitor [1,3].

As seen from the data in Table 1, like other coumarins investigated by our group these derivatives also do not inhibit the cytosolic isoforms hCA I and II up to 10 µM concentration of inhibitor in the assay system. hCA IV was also poorly inhibited, with most compounds being inactive whereas few of them showed activity in the high nanomolar range (e.g., **8a**, **8b** and **11a**, with K_I_s in the range of 350.4–848.3 nM). Several other coumarins, incuding **2a**, **3a** and **7b**, were micromolar hCA IV inhibitors, with K_I_s in the range of 2.65–8.48 µM. Thus, the 4-fluoroanilides of both 2-((2-oxo-2*H*-chromen-7-yl)oxy)acetic acid as well as its 6-isomer led to the best inhibitors of this isoform.

hCA IX on the other hand was effectively inhibited by most new coumarins reported here, except for **12a** and **12b** which were not hCA IX inhibitors up to 10 µM (Table 1). These compounds incorporate the morpholine-ethylamide moiety which is obviously inappropriate for obtaining effective CAIs in that position of the coumarin ring and with this type of substitution pattern. The remaining compounds showed an interesting hCA IX inhibitory patterns, with several compounds being quite effective inhibitors, with K_I_s in the range of 30.2–30.5 nM, similar to **AAZ** (K_I_ of 25 nM).

These compounds, **4b** and **5b**, are the phenethylamide and benzylamide derivatives of 2-((2-oxo-2*H*-chromen-6-yl)oxy)acetic acid **3b** and they are much more effective CA IX inhibitors compared to the corresponding 6-isomers **4a** and **5a** (Table 1). However, this was not always the case, as for other pairs of isoforms, the 7-isomer was a better hCA IX inhibitor compared to the corresponding 6-isomer (e.g., **2a**, which is a better inhibitor than **2b**; **11a**, a much more effective CA IX inhibitor compared to its isomer **11b**, etc.). Many other coumarins were slightly less effective hCA IX inhibitors, with K_I_s in the range of 83.7–290.2 nM. They include derivatives **2a**, **3a**, **3b**, **4a**, **5a**, **7a**, **7b**, **8a**, **8b**, **11a**, **13b** (Table 1). It is thus obvious that apart from the position in the coumarin ring where the substituent is appended, the most important factor influencing hCA IX inhibition is the nature of the moiety present on the amide part of the functionality. Indeed, the effective hCA IX inhibitors incorporate amides obtained from phenethylamine, benzylamine, aniline and substituted anilines. The only heterocyclic derivative leading to effective inhibitors was 4-pyridylmethylamine and piperidin-1-yl-ethylamine. The remaining amides (**2b**, **6a**, **6b**, **9a**, **9b**, **10a**, **10b**, **11b**, **13a**) were micromolar hCA IX inhibitors, with K_I_s in the range of 1.96–2.73 µM (Table 1). 

An important feature of many coumarins reported here is that they are highly selective hCA IX versus hCA I/II/IV inhibitors, and in many cases also very effective in inhibiting the tumor-associated isoform hCA IX without inhibition of the widespread cytosolic/membrane—bound isoforms I; II and IV. For example **4b** and **5b** are equipotent to acetazolamide as hCA IX inhibitors but do not inhibit at all hCA I, II and IV, whereas **AAZ** inhibits these three isoforms significantly (Table 1).

In vitro inhibition of soybean lipoxygenase (LOX) has also been investigated with the new coumarins reported here (Table 2). Eicosanoids are oxygenated metabolites of arachidonic acid with a broad implication in a diversity of diseases among which are included the pathogenesis of neutrophil-mediated inflammatory diseases with a marked relation to the severity of cardiovascular diseases, asthma and cancer [36]. 

In this context, we evaluated the synthesized compounds of Table 2 for their ability to inhibit soybean LOX by the UV absorbance based enzyme assay [34] using compounds samples with concentrations from 0.1–100 μM. Most of the LOX inhibitors are antioxidants or free radical scavengers. LOXs contain a non-heme iron per molecule in the enzyme active site as high-spin Fe^2+^ in the native state and the high spin Fe^3+^ in the activated state [35]. Some studies suggest a relationship between LOX inhibition and the ability of the inhibitors to reduce Fe^3+^ at the active site to the catalytically inactive Fe^2+^, whereas several LOX inhibitors are excellent ligands for Fe^3+^ [35]. Nordihydroguaiaretic acid (NDGA), a known inhibitor of soybean LOX, has been used as a reference compound (IC_50_ 0.45 μΜ/93% at 100 μM) and as a positive control in our experiments [35]. We determined the IC_50_ inhibition values for compounds **1a**, **3a**–**b**, **4a**–**b**, **6a**, **7a**, **9b**, **10a**, **11a**, **12a**–**b**, **13a**. We did not succeed to evaluate the IC_50_ values for the rest of the compounds, since they were not active LOX inhibitors at 100 μM (11–46%). The most potent % inhibition at 100 μM is shown by compound **4a** (**4a** > **4b** > **10a**~**11a** > **12b**~**6a**).

Perusal of the IC_50_’s inhibition values (Table 2) shows that the most potent, and equipotent, inhibitors are compounds **4a** and **4b** (10 μΜ) followed by **10a** (15 μΜ) and **12b** (16.5 μΜ). It is interesting to note that attachment on the coumarin ring, e.g., in the 6-/7- for compounds **4a** and **4b**, does not seem to play any role. Replacement of phenyl (**4a**) by a 2-pyridyl group (**10a**) or by a morpholinyl group (**12a**) leads to a reduction of the inhibitory activity, which is highly significant for **12a** (42.5 μΜ). The presence of a 2-pyridyl group in compound **10b** significantly decreased activity (by 40%) compared to **4b**. In a similar manner, the presence of a 4-pyridyl group (**11a**) resulted in significant loss of inhibitory activity (27 μΜ) compared to compound **10a**. The replacement by a morpholinyl group (**12b**) does not induce a considerable loss in activity.

The length of the chain between the aromatic ring and the NHCO-group [(CH_2_)_n_], influenced the biological response, since compound **4a** (10 μΜ) with n = 2, is more potent compared to **7a** (100 μΜ) in which n = 0 and **5a** (45%) in which n = 1. The same is seen for **7b** and **5b**. The F-substitution allows an improved inhibitory activity compared to the unsubstituted compound: for example **6a** has an IC_50_ of 47 µM, whereas **5a** only presents 45% at a concentration of 100 μΜ (Table 2). As concerns the acids **3a** and **3b** they appear to present some inhibitory activities (Table 2). Although lipophilicity is referred to as an important physicochemical property for LOX inhibitors [35], herein the theoretically calculated log P values did not always support this observation. The most potent compounds **4a** and **4b** showed the third higher lipophilicity values (2.60) in this series (Table 2). Furthermore, compounds with comparable lipophilicities showed in many cases striking different LOX inhibitory activities (Table 2).

## 3. Experimental Section

### 3.1. General Information

All biochemical reagents were of analytical grade and purchased from commercial sources. Soybean lipoxygenase, sodium linoleate, and NDGA were obtained from Sigma Chemical, Co. (St. Louis, MO, USA). 

### 3.2. Chemistry

#### 3.2.1. General Procedure for the Synthesis of Compounds **3a**–**b** [39]

A mixture of 7-hydroxycoumarin (**1a**) or 6-hydroxycoumarin (**1b**) (1 eq.) and potassium carbonate (3 eq.) was dissolved in dry DMF (5 mL) and the mixture was stirred at room temperature for 15 min. Then, ethyl 2-iodoacetate (**a**, 1.5 eq.) was added dropwise to the mixture under nitrogen atmosphere and heated to 100 °C for 30 min. After completion of the reaction (TLC monitoring) the mixture was cooled to room temperature and quenched with water and 1M aqueous HCl solution. The precipitated products **2a**–**b** were collected by filtration and washed with water, and used without further purification.


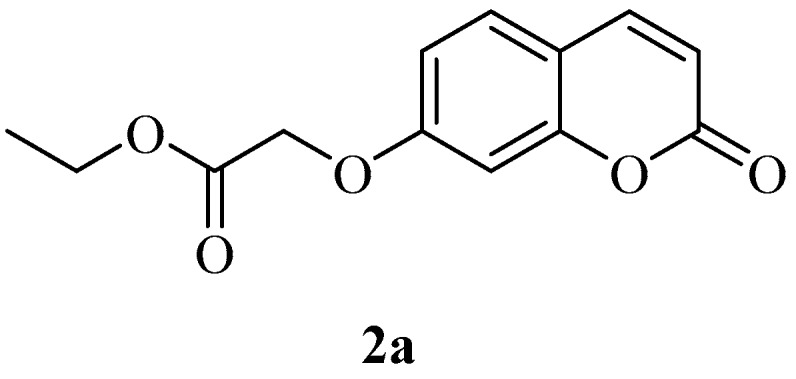


*Ethyl 2-((2-oxo-2H-chromen-7-yl)oxy)acetate* (**2a**). Using 7-hydroxycoumarin and, ethyl 2-iodoacetate as starting materials and the general procedure described above compound **2a** was obtained in 94% yield; m.p. 112.9–113.0 °C; δ_H_ (400 MHz, CDCl_3_) 1.31 (3H, t, *J* = 7.2 Hz), 4.28 (2H, q, *J* = 7.2 Hz), 4.68 (2H, s), 6.27 (1H, d, *J* = 9.5 Hz), 6.78 (1H, d, *J* = 2.4 Hz), 6.88 (1H, dd, *J* = 2.4, 8.6 Hz), 7.39 (1H, d, *J* = 8.6 Hz,), 7.63 (1H, d, *J* = 9.5 Hz); δ_C_ (100 MHz, CDCl_3_) 14.3, 61.9, 65.5, 101.9, 113.0, 113.5, 113.9, 129.1, 143.3, 155.8, 161.0, 161.0, 168.0; *m*/*z* (ESI positive) (C_13_H_12_O_5_) 249.2 [M + H]^+^. Experimental data are in agreement with the data reported in [40].


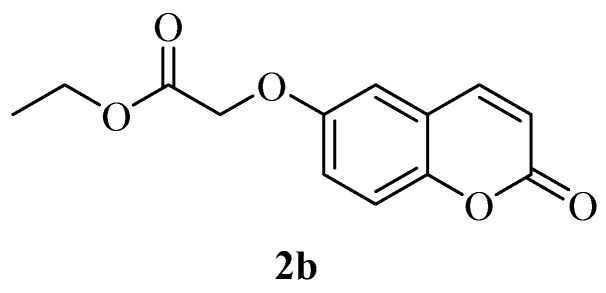


*Ethyl 2-((2-oxo-2H-chromen-6-yl)oxy)acetate* (**2b**)*.* Using 6-hydroxycoumarin and, ethyl 2-iodoacetate as starting materials and the general procedure described above compound 2a was obtained in 86% yield; m.p. 120–122 °C; δ_H_ (400 MHz, DMSO-*d*_6_) 1.26 (3H, t, *J* = 7.2 Hz), 4.21 (2H, q, *J* = 7.2 Hz), 4.88 (2H, s), 6.55(1H, d, *J* = 9.6 Hz), 7.27 (1H, dd, *J* = 3.0, 9.0 Hz), 7.33 (1H, d, *J* = 3.0 Hz), 7.39 (1H, d, *J* = 9.0 Hz), 8.03 (1H, d, *J* = 9.6 Hz); δ_C_ (100 MHz, DMSO-*d*_6_) 15.0, 61.6, 66.1, 112.9, 117.6, 118.3, 120.1, 120.6, 144.8, 149.2, 154.9, 161.0, 169.4; *m*/*z* (ESI positive) (C_13_H_12_O_5_) 249.2 [M + H]^+^. Experimental data are in agreement with those reported in [41].

The crude products **2a** or **2b** (2.7 mmol) were dissolved in an aqueous solution of 5% NaOH (5 mL) in ethanol (15 mL) and the mixture was stirred at room temperature for 5 min. The residue quenched with water and acidified with aqueous 6 M solution HCl. The precipitated white solid was filtered off and subsequently washed with cool water and DCM to give compounds **3a**–**b**, respectively.

*2-((2-Oxo-2H-chromen-7-yl)oxy)acetic acid* (**3a**). Compound **3a** was obtained in 98% yield; m.p. 180–182 °C; δ_H_ (400 MHz, DMSO-*d*_6_) 4.86 (2H, s), 6.34 (1H, d, *J* = 9.2 Hz), 7.98–7.00 (2H, m), 7.68 (1H, d, *J* = 9.6 Hz,), 8.03 (1H, d, *J* = 9.2 Hz); δ_C_ (100 MHz, DMSO-*d*_6_) 65.8, 102.4, 113.5, 113.7, 113.7, 130.4, 145.2, 156.1, 161.2, 161.8, 170.5; *m*/*z* (ESI positive) (C_11_H_8_O_5_) 221.0 [M + H]^+^. Experimental data are in agreement with those reported in [40]. 

*2-((2-Oxo-2H-chromen-6-yl)oxy)acetic acid* (**3b**). Compound **3b** was obtained in 87% yield; m.p. 163.5–163.7 °C; δ_H_ (400 MHz, DMSO-*d*_6_) 4.78 (2H, s,), 6.53(1H, d, *J* = 9.5 Hz), 7.25 (1H, dd, *J* = 3.0, 9.0 Hz), 7.30 (1H, d, *J* = 3.0 Hz,), 7.38 (1H, d, *J* = 9.0 Hz), 8.03 (1H, d, *J* = 9.5 Hz); δ_C_ (100 MHz, DMSO-*d*_6_) 65.9, 112.7, 117.6, 118.2, 120.1, 120.6, 144.9, 149.0, 155.0, 160.1, 170.8; *m*/*z* (ESI positive) 221. [M + H]^+^, *m*/*z* (ESI negative) 219.0 [M − H]^−^. Experimental data are in agreement with those reported in [41].

#### 3.2.2. General Procedure for the Synthesis of **4a**,**b**–**13a**,**b**

The appropriate coumarin acetic acid derivative 2-((2-oxo-2*H*-chromen-7-yl)oxy)acetic acid (**3a**) or 2-((2-oxo-2*H*-chromen-6-yl)oxy)acetic acid (**3b**) (1.0 eq.), 1-ethyl-3-(3-dimethylaminopropyl) carbodiimide hydrochloride salt (EDCI·HCl, 1.5 eq.) and 1-hydroxy-7-azabenzotriazole (HOAT, 1.5 eq.), were dissolved in dry DMA (3.0 mL) and stirred for 10 min at r.t., followed by addition of the corresponding amine **4**–**13** (1.0 eq.) and *N*,*N*-diisopropylethylamine (DIPEA, 4.0 eq.) or triethylamine (Et_3_N, 5.0 eq.) in the same solvent (2.0 mL). The reaction mixture was stirred until the consumption of starting materials (TLC monitoring) and quenched with water and 6.0 M aqueous HCl solution at 0–5 °C. The crude products were collected by filtration and washed with cool water, DCM and diethyl ether to obtain desired products **4a**,**b**–**13a**,**b**.


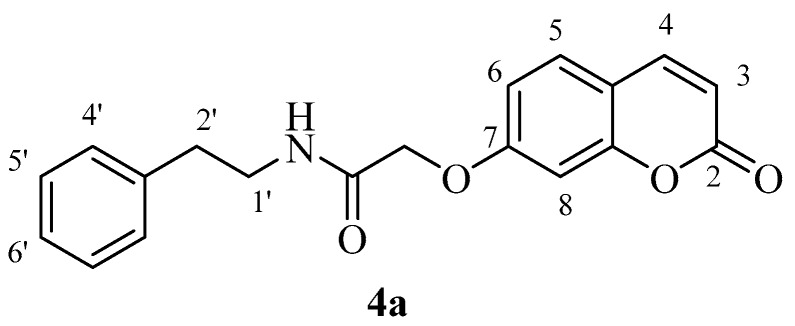


*2-((2-Oxo-2H-chromen-7-yl)oxy)-N-phenethylacetamide* (**4a**). Using **3a** and **4** as starting materials and the general procedure described above compound **4a** was obtained as a white solid in 80% yield; m.p. 178–179 °C; silica gel TLC *R_f_* = 0.73 (MeOH/DCM 10% *v*/*v*); IR (KBr, cm^−1^) 1710.1, 1604.1, 1558.2; δ_H_ (400 MHz, DMSO-*d*_6_) 2.79 (2H, t, *J* = 7.2 Hz, 2′-H_2_), 3.39 (2H, q, *J* = 7.2 Hz, 1′-H_2_), 4.63 (2H, s, OC*H*_2_CO), 6.35 (1H, d, *J =* 9.5 Hz, 3-H), 7.00 (2H, m, 6, 8-H), 7.20–7.24 (3H, m, 4′, 6′-H), 7.29–7.33 (2H, m, 5′-H), 7.68 (1H, d, *J* = 8.6 Hz, 5-H), 8.04 (1H, d, *J* = 9.5 Hz, 4-H), 8.27 (1H, t, *J =* 7.2 Hz, exchangeable with D_2_O, N*H*); δ_C_ (100 MHz, DMSO-*d*_6_) 35.9, 68.1, 102.6, 113.6, 113.7, 113.8, 127.0, 129.2, 129.5, 130.4, 140.1, 145.1, 156.0, 161.0, 161.7, 167.7; *m*/*z* (ESI positive) (C_19_H_17_NO_4_) 324.3 [M + H]^+^.


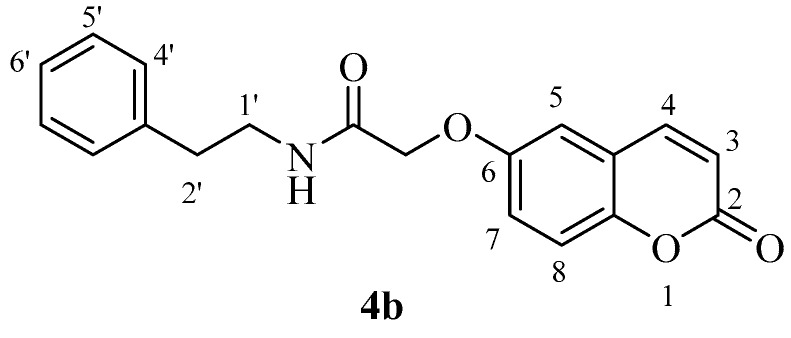


*2-((2-Oxo-2H-chromen-6-yl)oxy)-N-phenethylacetamide* (**4b**). Using **3b** and **4** as starting materials and the general procedure described above compound **4b** was obtained as a white solid 64% yield; m.p. 150–151°C; silica gel TLC *R_f_ =* 0.5 (MeOH/DCM 10% *v*/*v*); IR (KBr, cm^−1^) 1699.2, 1670.5, 1610.1, 1560.2; δ_H_ (400 MHz, DMSO-*d*_6_) 2.74 (2H, t, *J* = 7.2 Hz, 2′-H_2_), 3.39 (2H, q, *J* = 7.2 Hz, 1′-H_2_), 4.50 (2H, s, OC*H*_2_CO), 6.52 (1H, d, *J =* 9.6 Hz, 3-H), 7.20–7.32 (7H, m, 5, 7, 4′, 5′, 6′-H), 7.40 (1H, d, *J* = 8.8, 8-H), 8.03 (1H, d, *J* = 9.6 Hz, 4-H), 8.21 (1H, t, *J =* 7.2 Hz, exchangeable with D_2_O, N*H*); δ*_C_* (100 MHz, DMSO-*d*_6_) 35.9, 40.8, 68.4, 112.9, 117.6, 118.2, 120.0, 120.9, 127.0, 129.2, 129.5, 140.2, 144.9, 149.1, 154.9, 160.9, 168.1; *m*/*z* (ESI positive) (C_19_H_17_NO_4_) 324.3 [M + H]^+^.


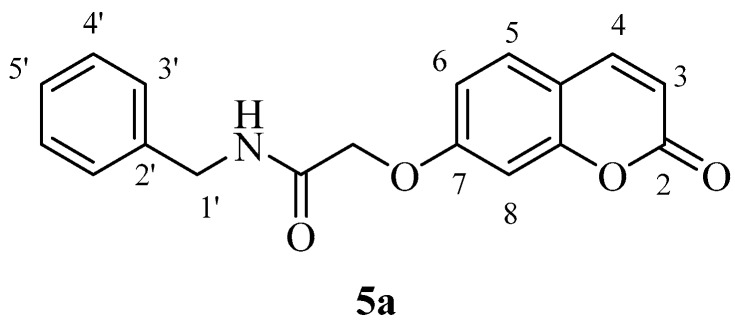


*N-Benzyl-2-((2-oxo-2H-chromen-7-yl)oxy)acetamide* (**5a**)*.* Using **3a** and **5** as starting materials and the general procedure described above compound **5a** was obtained as a white solid 67% yield; m.p. 167–168 °C; silica gel TLC *R_f_ =* 0.52 (MeOH/DCM 10% *v*/*v*); IR (KBr, cm^−1^) 1699.5, 1672.7, 1612.8, 1550.4; δ_H_ (400 MHz, DMSO-*d*_6_) 4.40 (2H, d, *J* = 6.1 Hz, 1′-H), 4.74 (2H, s, OC*H*_2_CO), 6.35 (1H, d, *J =* 9.5 Hz, 3-H), 7.03–7.06 (2H, m, 6, 8-H), 7.25–7.36 (5H, m, 3′, 4′, 5′-H), 7.69 (1H, d, *J* = 8.4 Hz, 5-H), 8.03 (1H, d, *J* = 9.6 Hz, 4-H), 8.78 (1H, t, *J =* 6.1 Hz, exchange with D_2_O, N*H*); δ_C_ (100 MHz, DMSO-*d*_6_) 42.8, 68.2, 102.7, 113.7, 113.8, 113.9, 127.8, 128.2, 129.2, 130.5, 140.2, 145.2, 156.1, 161.2, 161.7, 168.1; *m*/*z* (ESI positive) (C_18_H_15_NO_4_) 310.2 [M + H]^+^. 


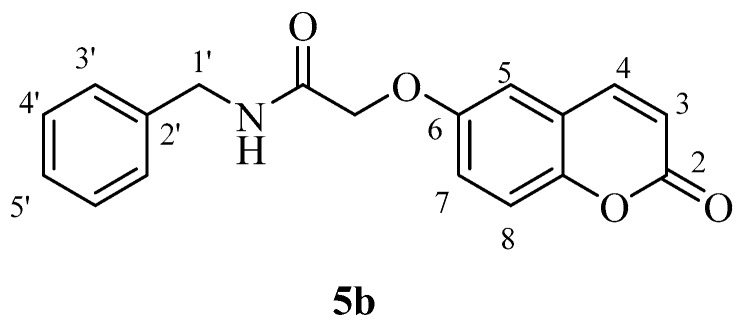


*N-Benzyl-2-((2-oxo-2H-chromen-6-yl)oxy)acetamide* (**5b**). Using **3b** and **5** as starting materials and the general procedure described above compound **5b** was obtained as a white solid in 67% yield; m.p. 161–162 °C; silica gel TLC *R_f_ =* 0.63 (MeOH/DCM 10% *v*/*v*); IR (KBr, cm^−1^) 1705.2, 1680.5, 1605.7, 1555.2; δ_H_ (400 MHz, DMSO-*d*_6_) 4.38 (2H, d, *J* = 6.2 Hz, 1′-H), 4.65 (2H, s, O*CH*_2_CO), 6.52 (1H, d, *J =* 9.6 Hz, 3-H), 7.25–7.36 (7H, m, 3′, 4′, 5′-H), 7.40 (1H, d, *J* = 8.6 Hz, 8-H), 8.02 (1H, d, *J* = 9.6 Hz, 4-H), 8.07 (1H, t, *J* = 6.2 Hz, exchange with D_2_O, N*H*); δ_C_ (100 MHz, DMSO-*d*_6_) 42.9, 68.5, 113.0, 117.7, 118.4, 120.2, 121.1, 127.8, 128.2, 129.2, 140.3, 145.2, 149.2, 155.1, 161.1, 168.6; *m*/*z* (ESI positive) (C_18_H_15_NO_4_) 310.2 [M + H]^+^.


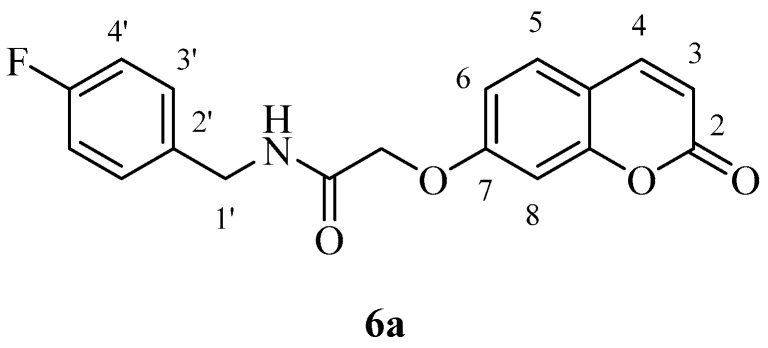


*N-(4-Fluorobenzyl)-2-((2-oxo-2H-chromen-7-yl)oxy)acetamide* (**6a**). Using **3a** and **6** as starting materials and the general procedure described above compound **6a** was obtained as a white solid in 54% yield; m.p. 160–161 °C; silica gel TLC *R_f_ =* 0.62 (MeOH/DCM 10% *v*/*v*); IR (KBr, cm^−1^) 1711.6, 1673.1, 1554.8, 1549.6; δ_H_ (400 MHz, DMSO-*d*_6_) 4.37 (2H, d, *J* = 6 Hz, 1′-H), 4.73 (2H, s, O*CH*_2_CO), 6.36 (1H, d, *J =* 9.6 Hz, 3-H), 7.06–7.10 (2H, m, 6, 8-H), 7.18–7.23 (2H, m, 4′-H), 7.67–7.71 (3H, m, 5, 3′-H), 7.69 (1H, d, *J* = 8.4 Hz), 8.04 (1H, d, *J* = 9.6 Hz, 4-H), 8.77 (1H, t, *J* = 6 Hz, exchange with D_2_O, N*H*); δ_C_ (100 MHz, DMSO-*d*_6_) 42.1, 68.1, 102.6, 113.7, 113.8, 113.8, 115.8 (d, ^2^*J*_C-F_ 22), 130.2 (d, ^3^*J*_C-F_ 8), 130.4, 136.3 (d, ^4^*J*_C-F_ 3), 145.2, 156.0, 161.7, 162.1 (d, ^1^*J*_C-F_ 238.8), 161.61, 168.0; δ_F_ (376 MHz, DMSO-*d*_6_) −115.9 (1F, s); *m*/*z* (ESI positive) (C_18_H_14_FNO_4_) 328.2 [M + H]^+^. 


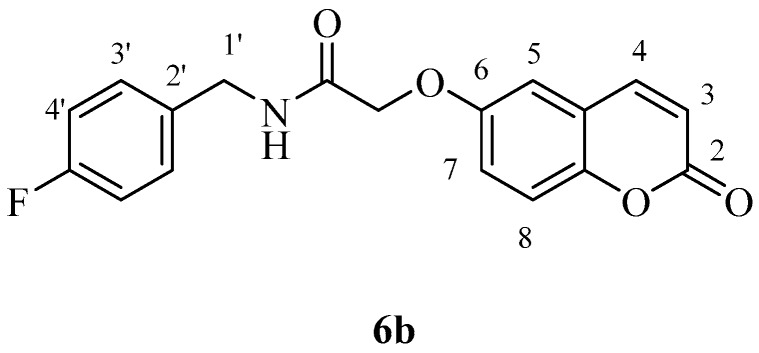


*N-(4-Fluorobenzyl)-2-((2-oxo-2H-chromen-6-yl)oxy)acetamide* (**6b**). Using **3b** and **6** as starting materials and the general procedure described above compound **6b** was obtained as a white solid in 45% yield; m.p. 158–159 °C; silica gel TLC *R_f_ =* 0.42 (MeOH/DCM 10% *v*/*v*); IR (KBr, cm^−1^) 1698.6, 1672.3, 1615.0, 1554.9; δ_H_ (400 MHz, DMSO-*d*_6_) 4.36 (2H, d, *J* = 6 Hz, 1′-H), 4.65 (2H, s, O*CH*_2_CO), 6.54 (1H, d, *J =* 9.6 Hz, 3-H), 7.17–7.21 (2H, m, 4′-H), 7.33–7.42 (3H, m, 5, 7, 8-H), 7.67–7.70 (2H, m, 3′-H), 8.04 (1H, d, *J* = 9.6 Hz, 4-H), 8.76 (1H, t, *J* = 6 Hz, exchange with D_2_O, N*H*); δ_C_ (100 MHz, DMSO-*d*_6_) 42.0, 68.4, 112.9, 115.8 (d, ^2^*J*_C-F_ 21), 117.6, 118.3, 120.0, 121.0, 130.2 (d, ^3^*J*_C-F_ 8), 136.4 (d, ^4^*J*_C-F_ 3), 144.9, 149.1, 154.9, 161.0, 162.06 (d, ^1^J_C-F_ 240), 168.4. δ_F_ (376 MHz, DMSO-*d*_6_) −116.1 (1F, s); *m*/*z* (ESI positive) (C_18_H_14_FNO_4_) 328.2 [M + H]^+^.


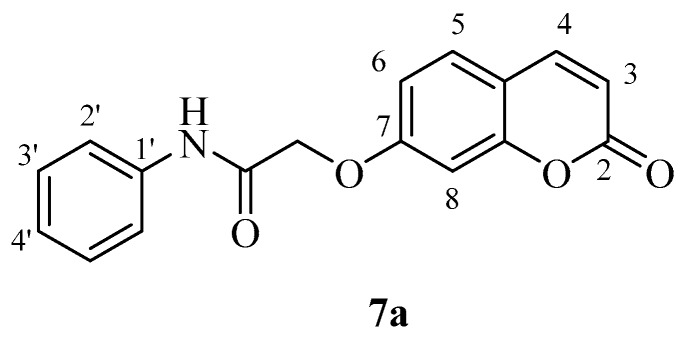


*2-((2-Oxo-2H-chromen-7-yl)oxy)-N-phenylacetamide* (**7a**)*.* Using **3a** and **7** as starting materials and the general procedure described above compound **7a** was obtained as a white solid in 70% yield; m.p. 182–183 °C; silica gel TLC *R_f_ =* 0.57 (MeOH/DCM 10% *v*/*v*); δ_H_ (400 MHz, DMSO-*d*_6_) 4.96 (2H, s, CO*CH*_2_O), 6.35 (1H, d, *J =* 8 Hz, 3-H), 7.10 (3H, m), 7.36 (2H, m), 7.69 (3H, m), 8.04 (1H, d, *J* = 8 Hz), 10.22 (1H, s, exchange with D_2_O, N*H*); δ_C_ (100 MHz, DMSO-*d*_6_) 68.2, 102.6, 113.6, 113.8, 113.9, 120.6, 124.7, 129.6, 130.4, 139.2, 145.1, 156.0, 161.1, 161.9, 166.7; *m*/*z* (ESI positive) (C_17_H_13_NO_4_) 296.2 [M + H]^+^. Experimental data are in agreement with those reported in [39].


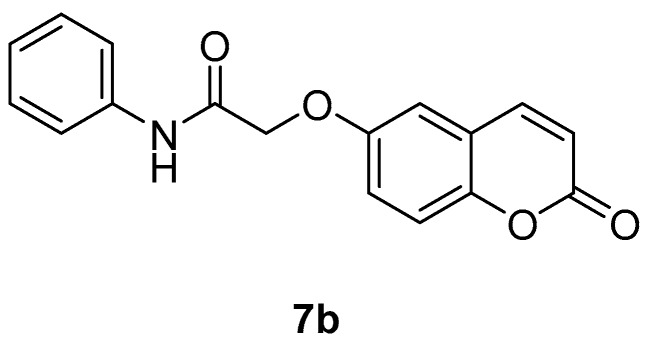


*2-((2-Oxo-2H-chromen-6-yl)oxy)-N-phenylacetamide* (**7b**) [39]. Using **3b** and **7** as starting materials and the general procedure described above compound **7b** was obtained as a white solid in 80% yield; m.p. 182–183 °C; silica gel TLC *R_f_* 0.56 (MeOH/DCM 10% *v*/*v*); δ_H_ (400 MHz, DMSO-*d*_6_) 4.80 (2H, s), 6.54 (1H, d, *J =* 9.6 Hz), 7.12 (1H, t*, J =* 7.4 Hz), 7.36 (4H, m), 7.43 (1H, d, *J* = 8.7), 7.68 (2H, d, *J* = 7.4 Hz), 8.07 (1H, d, *J* = 9.6 Hz), 10.14 (1H, s, exchange with D_2_O, N*H*); δ_C_ (100 MHz, DMSO-*d*_6_) 68.6, 113.0, 117.6, 118.3, 120.1, 120.6, 120.8, 124.6, 129.7, 139.2, 144.9, 149.1, 155.1, 161.0, 167.1. *m*/*z* (ESI positive) (C_17_H_13_NO_4_) 296.2 [M + H]^+^. Experimental data are in agreement with those reported in [39].


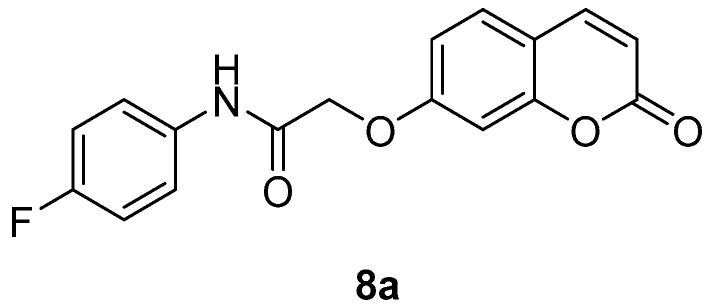


*N-(4-Fluorophenyl)-2-((2-oxo-2H-chromen-7-yl)oxy)acetamide* (**8a**) [39]. Using **3a** and **8** as starting materials and the general procedure described above compound **8a** was obtained as a white solid in 80% yield; m.p. 208–209 °C; silica gel TLC *R_f_* 0.75 (MeOH/DCM 10% *v*/*v*); δ_H_ (400 MHz, DMSO-*d*_6_) 4.87 (2H, s), 6.35 (1H, d, *J =* 9.5 Hz), 7.08 (2H, m), 7.21 (2H, m), 7.69 (3H, m), 8.03 (1H, d, *J* = 9.5 Hz), 10.25 (1H, s, exchange with D_2_O, N*H*); δ_C_ (100 MHz, DMSO-*d*_6_) 68.2, 102.6, 113.7, 113.9, 113.9, 116.4 (d, ^2^*J*_C-F_ = 22), 122.6 (d, ^3^*J*_C-F_ = 8), 130.5, 135.6 (d, ^4^*J*_C-F_ = 3), 145.2, 156.1, 159.3 (d, ^1^*J*_C-F_ = 239), 161.2, 161.9, 166.8; δ_F_ (376 MHz, DMSO-*d*_6_) −118.7 (1F, s); *m*/*z* (ESI positive) (C_17_H_12_FNO_4_) 314.2 [M + H]^+^. Experimental data are in agreement with those reported in [39].


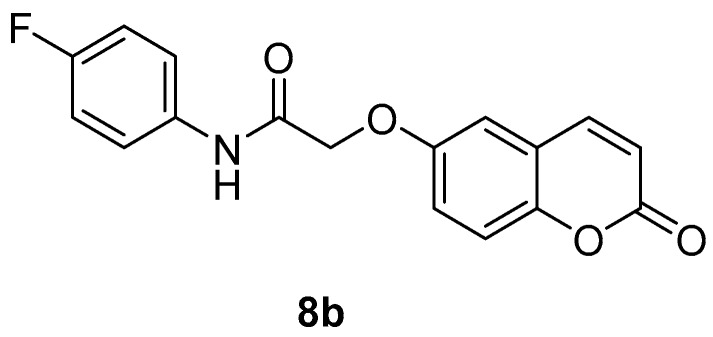


*N-(4-Fluorophenyl)-2-((2-oxo-2H-chromen-6-yl)oxy)acetamide* (**8b**) [39]. Using **3b** and **8** as starting materials and the general procedure described above compound **8b** was obtained as a white solid in 80% yield; m.p. 206–207 °C; silica gel TLC *R_f_* 0.86 (MeOH/DCM 10% *v*/*v*); δ_H_ (400 MHz, DMSO-*d*_6_) 4.79 (2H, s), 6.52 (1H, d, *J =* 9.6 Hz), 7.18 (2H, t, *J* = 8.8), 7.38 (3H, m), 7.68 (2H, m), 8.03 (1H, d, *J* = 9.6 Hz), 10.22 (1H, s, exchange with D_2_O, N*H*); δ*_C_* (100 MHz, DMSO-*d*_6_) 68.6, 113.1, 116.3 (d, ^2^*J*_C-F_ 22), 117.7, 118.5, 120.2, 121.0, 122.8 (d, ^3^*J*_C-F_ 8), 135.6 (d, ^4^*J*_C-F_ 3), 145.1, 149.3, 155.2, 159.3 (d, ^1^*J*_C-F_ 238.8), 161.2, 167.3. δ_F_ (376 MHz, DMSO-*d*_6_) −118.78 (1F, s); *m*/*z* (ESI positive) (C_17_H_12_FNO_4_) 314.2 [M + H]^+^. Experimental data are in agreement with those reported in [39].


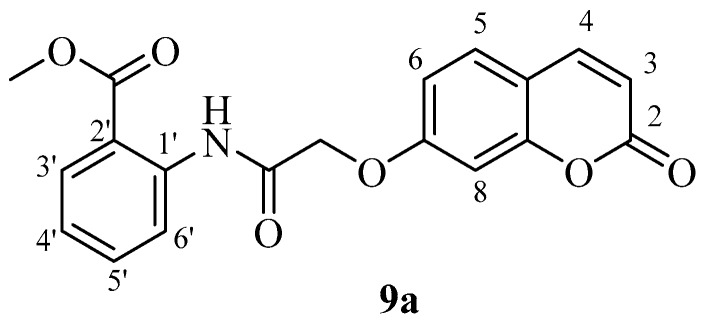


*Methyl 2-(2-((2-oxo-2H-chromen-7-yl)oxy)acetamido)benzoate* (**9a**). Using **3a** and **9** as starting materials and the general procedure described above compound **9a** was obtained as a white solid in 45% yield; m.p. 213–214 °C; silica gel TLC *R_f_ =* 0.69 (MeOH/DCM 10% *v*/*v*); IR (KBr, cm^−1^) 1710.3, 1668.7, 1612.3, 1562.0; δ_H_ (400 MHz, DMSO-*d*_6_) 3.96 (3H, s, COO*CH*_3_), 4.93 (2H, s, OC*H*_2_CO), 6.39 (1H, d, *J =* 9.6 Hz, 3-H), 7.15-7.20 (2H, m, 6, 8-H), 7.28 (1H, t, *J* = 7.5, 4′-H), 7.70 (1H, t, *J* = 7.5, 5′-H), 7.76 (1H, d, *J* = 9.2, 5-H), 8.03-8.09 (2H, m, 4, 6′-H), 8.62 (1H, d, *J* = 7.5 Hz, 3′-H), 11.71 (1H, s, exchange with D_2_O, N*H*); δ*_C_* (100 MHz, DMSO-*d*_6_) 53.5, 68.5, 102.8, 113.7, 114.1, 114.2, 117.2, 121.1, 124.4, 130.7, 131.7, 135.4, 140.3, 145.1, 156.1, 160.9, 161.0, 167.3, 168.4. *m*/*z* (ESI positive) (C_19_H_15_NO_6_) 354 [M + H]^+^.


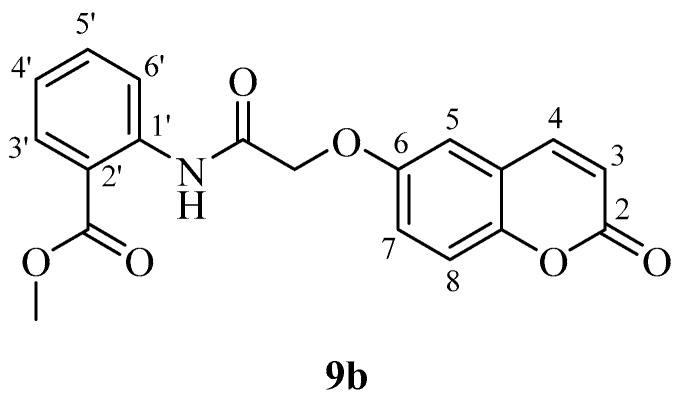


*Methyl 2-(2-((2-oxo-2H-chromen-6-yl)oxy)acetamido)benzoate* (**9b**). Using **3b** and **9** as starting materials and the general procedure described above compound **9b** was obtained as a white solid in 50% yield; m.p. 213–214 °C; silica gel TLC *R_f_ =* 0.67 (MeOH/DCM 10% *v*/*v*); IR (KBr, cm^−1^) 1712.9, 1670.4, 1611.9, 1563.8; δ_H_ (400 MHz, DMSO-*d*_6_) 3.94 (3H, s, COO*CH*_3_), 4.85 (2H, s, O*CH*_3_CO), 6.56 (1H, d, *J =* 9.6 Hz, 3-H), 7.27 (1H, t, *J* = 7.8, 4′-H), 7.43 (1H, dd, *J* 2.8, 9.1 Hz, 7-H), 7.47-7.50 (2H, m, 5, 8-H), 7.71 (1H, t, *J* = 7.8, 5′-H), 8.05 (1H, d, *J* = 7.8, 6′-H), 8.08 (1H, d, *J* = 9.6 Hz, 4-H), 8.65 (1H, d, *J* = 7.8 Hz, 3′-H), 11.74 (1H, s, exchange with D_2_O, N*H*); δ*_C_* (100 MHz, DMSO-*d*_6_) 51.6, 66.2, 110.4, 113.2, 113.9, 115.6, 118.3, 119.4, 119.5, 124.2, 130.3, 133.6, 141.3, 143.5, 147.8, 157.6, 163.2, 167.7, 168.3; *m*/*z* (ESI negative) (C_19_H_15_NO_6_) 352 [M − H]^−^.


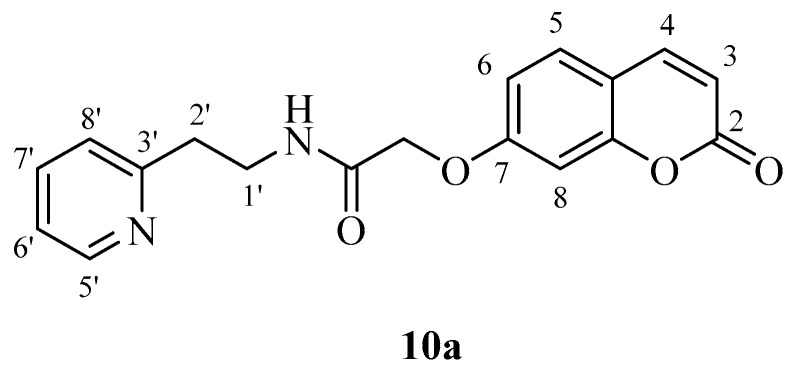


*2-((2-Oxo-2H-chromen-7-yl)oxy)-N-(2-(pyridin-2-yl)ethyl)acetamide* (**10a**). Using **3a** and **10** as starting materials and the general procedure described above compound **10a** was obtained as a white solid in 68% yield; m.p. 138–139 °C; silica gel TLC *R_f_* = 0.60 (MeOH/DCM 10% *v*/*v*); IR (KBr, cm^−1^) 1710.5, 1672.3, 1612.8, 1554.7; δ_H_ (400 MHz, DMSO-*d*_6_) 2.95 (2H, t, *J* = 7.2 Hz, 2′-H), 3.54 (2H, q, *J* = 7.2 Hz, 1′-H), 4.63 (2H, s, O*CH*_2_CO), 6.36 (1H, d, *J =* 9.6 Hz, 3-H), 6.97 (1H, d, *J* = 2.4 Hz, 8-H), 7.00 (1H, dd, *J* = 2.4, 8.6 Hz, 6-H), 7.22–7.24 (1H, m, 6′-H), 7.26 (1H, d, *J* = 8.6 Hz, 5-H), 7.67–7.73 (2H, m, 5′,8′-H), 8.03 (1H, d, *J* = 9.6 Hz, 4-H), 8.27 (1H, t, *J* = 7.2 Hz, exchange with D_2_O, N*H*), 8.50-8.52 (1H, m, 7′-H); δ*_C_* (100 MHz, DMSO-*d*_6_) 38.2, 39.8, 68.5, 103.2, 114.4, 114.5, 114.6, 123.5, 125.1, 131.3, 138.7, 146.2, 150.4, 156.6, 160.2, 162.3, 162.6, 169.4; *m*/*z* (ESI positive) (C_18_H_16_N_2_O_4_) 325.3 [M + H]^+^.


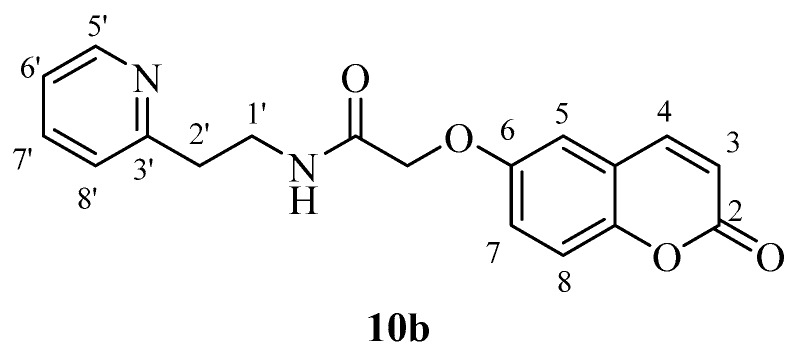


*2-((2-Oxo-2H-chromen-6-yl)oxy)-N-(2-(pyridin-2-yl)ethyl)acetamide* (**10b**). Using **3b** and **10** as starting materials and the general procedure described above compound **10b** was obtained as a white solid in 60% yield; m.p. 141–142 °C; silica gel TLC *R_f_* = 0.60 (MeOH/DCM 10% *v*/*v*); IR (KBr, cm^−1^) 1700.8, 1671.5, 1609.1, 1556.8; δ_H_ (400 MHz, DMSO-*d*_6_) 2.95 (2H, t, *J* = 6.4 Hz, 2′-H), 3.55 (2H, q, *J* = 6.4 Hz, 1′-H), 4.56 (2H, s, O*CH*_2_CO), 6.54 (1H, d, *J =* 9.6 Hz, 3-H), 7.21-7.30 (4H, m, 7, 5, 6′, 8′-H), 7.39 (1H, d, *J* = 8.8 Hz, 8-H), 7.70 (1H, t, *J* = 7.8 Hz, 7′-H), 8.04 (1H, d, *J* = 9.6 Hz, 4-H), 8.27 (1H, t, *J* = 6.4 Hz, exchange with D_2_O, N*H*), 8.51 (1H, m, 5′-H); δ_C_ (100 MHz, DMSO-*d*_6_) 36.4, 54.1, 67.0, 112.9, 113.4, 113.9, 117.6, 118.3, 120.1, 120.9, 136.9, 143.7, 144.9, 149.1, 154.9, 158.7, 160.9, 168.1; *m*/*z* (ESI positive) (C_18_H_16_N_2_O_4_) 325.3 [M + H]^+^.


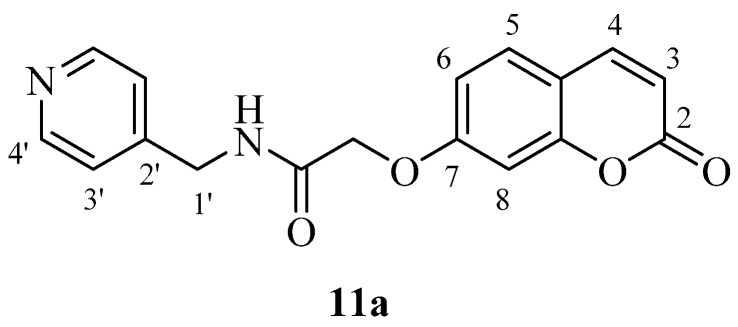


*2-((2-Oxo-2H-chromen-7-yl)oxy)-N-(pyridin-4-ylmethyl)acetamide* (**11a**). Using **3a** and **11** as starting materials and the general procedure described above compound **11a** was obtained as a white solid in 62% yield; m.p. 158–159 °C; silica gel TLC *R_f_* = 0.43 (MeOH/DCM 10% *v*/*v*); IR (KBr, cm^−1^) 1699.7, 1670.3, 1605.2, 1555.1; δ_H_ (400 MHz, DMSO-*d*_6_) 4.42 (2H, d, *J* = 6.1 Hz, 1′-H), 4.70 (2H, s, O*CH*_2_CO), 6.55 (1H, d, *J =* 9.6 Hz, 3-H), 7.27–7.29 (2H, 6, 8-H), 7.33–7.36 (2H, m, 3′-H), 7.42 (1H, d, *J* = 8.9 Hz, 5-H), 8.05 (1H, d, *J* = 9.6 Hz, 4-H), 8.50–8.52 (2H, m, 4′-H), 8.85 (1H, t, *J* = 6.1 Hz, exchange with D_2_O, N*H*); δ*_C_* (100 MHz, DMSO-*d*_6_) 41.8, 68.3, 112.9, 117.6, 118.3, 120.0, 121.0, 123.0, 144.8, 149.1, 149.2, 150.3, 154.9, 160.9, 168.8; *m*/*z* (ESI positive) (C_17_H_14_N_2_O_4_) 311.2 [M + H]^+^.


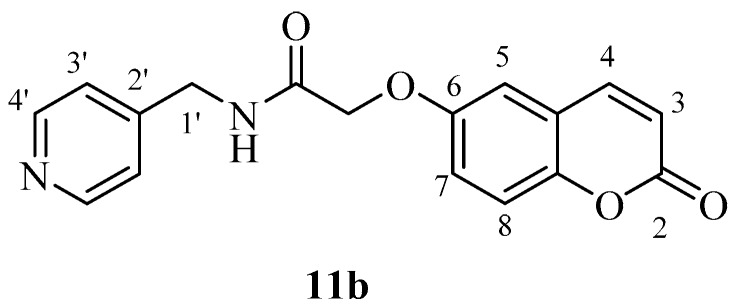


*2-((2-Oxo-2H-chromen-6-yl)oxy)-N-(pyridin-4-ylmethyl)acetamide* (**11b**). Using **3b** and **11** as starting materials and the general procedure described above compound **11b** was obtained as a white solid in 62% yield; m.p. 160–161 °C; silica gel TLC *R_f_* = 0.40 (MeOH/DCM 10% *v*/*v*); IR (KBr, cm^−1^) 1709.3, 1671.6, 1606.5, 1555.0; δ_H_ (400 MHz, DMSO-*d*_6_) 4.41 (2H, d, *J* = 6.1 Hz, 1′-H), 4.78 (2H, s, O*CH*_2_CO), 6.36 (1H, d, *J =* 9.6 Hz, 3-H), 7.05–7.07 (2H, m, 6, 8-H), 7.27–7.30 (2H, m, 3′-H), 7.70 (1H, d, *J =* 8.6 Hz, 8-H), 8.06 (1H, d, *J* = 9.6 Hz, 4-H), 8.51-8.53 (2H, m, 4′-H), 8.85 (1H, t, *J* = 6.1 Hz, exchange with D_2_O, N*H*); δ*_C_* (100 MHz, DMSO-*d*_6_) 41.8, 68.1, 102.7, 113.7, 113.8, 113.9, 123.0, 130.4, 145.1, 149.1, 150.4, 156.1, 157.3, 161.6, 168.4; *m*/*z* (ESI positive) (C_17_H_14_N_2_O_4_) 311.2 [M + H]^+^. 


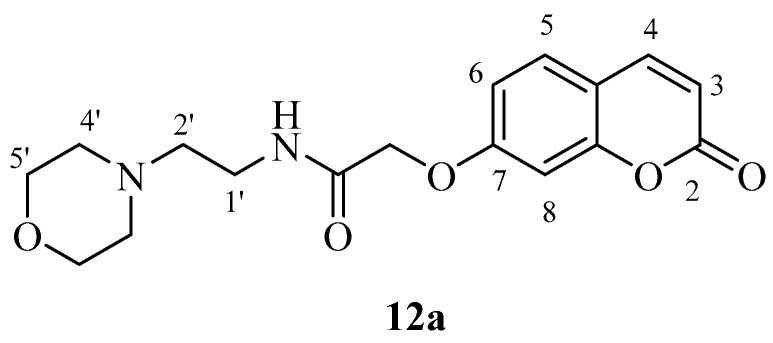


*N-(2-Morpholinoethyl)-2-((2-oxo-2H-chromen-7-yl)oxy)acetamide* (**12a**)*.* Using **3a** and **12** as starting materials and the general procedure described above compound **12a** was obtained as a white solid in 30% yield; m.p. 148–149 °C; silica gel TLC *R_f_* = 0.50 (MeOH/DCM 10% *v*/*v*); IR (KBr, cm^−1^) 1712.7, 1671.9, 1607.3, 1553.8; δ_H_ (400 MHz, DMSO-*d*_6_) 2.38–2.43 (6H, m, 2′, 4′-H), 3.30 (2H, q, *J* = 6.3 Hz, 1′-H), 3.57 (4H, t, *J* = 4.7 Hz, 5′-H), 4.66 (2H, s, O*CH*_2_CO), 6.36 (1H, d, *J =* 9.6 Hz, 3-H), 7.00–7.05 (2H, m, 6, 8-H), 7.70 (1H, d, *J* = 8.5 Hz, 5-H), 8.05 (1H, d, *J =* 9.6 Hz, 4-H), 8.09 (1H, t, *J* = 6.3 Hz, exchange with D_2_O, N*H*); δ*_C_* (100 MHz, DMSO-*d*_6_) 39.8, 54.1, 58.0, 67.0, 68.1, 102.6, 113.7, 113.8, 113.9, 130.4, 145.1, 156.0, 161.0, 161.6, 167.7; *m*/*z* (ESI positive) (C_17_H_20_N_2_O_5_) 333.3 [M + H]^+^.


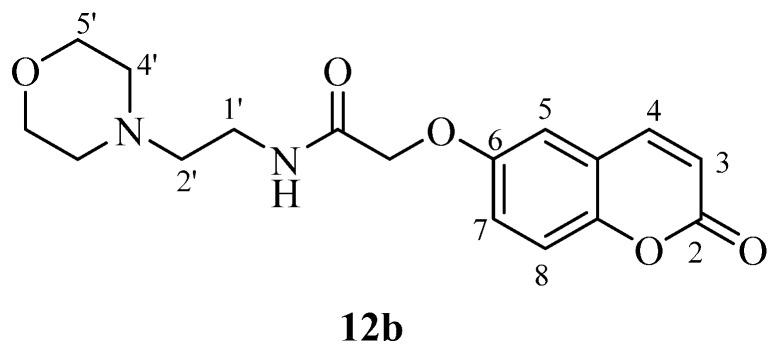


*N-(2-Morpholinoethyl)-2-((2-oxo-2H-chromen-6-yl)oxy)acetamide* (**12b**). Using **3b** and **12** as starting materials and the general procedure described above compound **12b** was obtained as a white solid in 30% yield; m.p. 149–150 °C; silica gel TLC *R_f_* = 0.57 (MeOH/DCM 10% *v*/*v*); IR (KBr, cm^−1^) 1700.2, 1678.4, 1617.2, 1557.0; δ_H_ (400 MHz, DMSO-*d*_6_) 2.37–2.43 (6H, m, 2′, 4′-H), 3.30 (2H, q, *J* = 6.6 Hz, 1′-H), 3.56 (4H, t, *J* = 4.6 Hz, 5′-H), 4.58 (2H, s, O*CH*_2_CO), 6.54 (1H, d, *J =* 9.6 Hz, 3-H), 7.30 (1H, dd, *J* = 2.8, 8.9 = Hz, 7-H), 7.33 (1H, d, *J* = 2.8, 5-H), 7.42 (1H, d, *J* = 8.9 Hz, 8-H), 8.01 (1H, t, *J* = 6.6 Hz, exchange with D_2_O, N*H*), 8.05 (1H, d, *J =* 9.6 Hz, 4-H); δ*_C_* (100 MHz, DMSO-*d*_6_) 39.6, 55.8, 58.9, 66.7, 67.0, 112.9, 113.4, 113.9, 118.1, 120.0, 143.5, 145.3, 156.3, 160.8, 168.6; *m*/*z* (ESI positive) (C_17_H_20_N_2_O_5_) 333.3 [M + H]^+^.


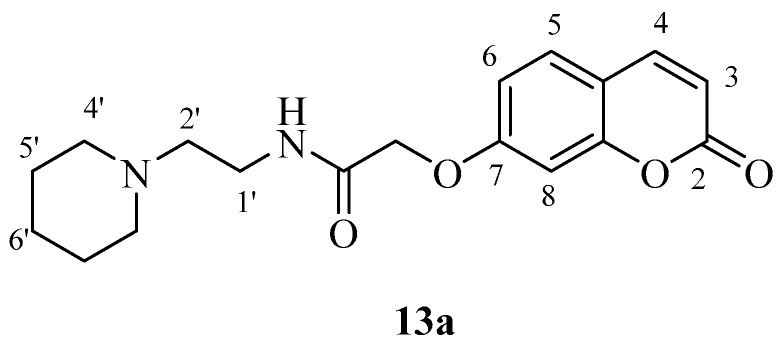


*2-((2-Oxo-2H-chromen-7-yl)oxy)-N-(2-(piperidin-1-yl)ethyl)acetamide* (**13a**). Using **3a** and **13** as starting materials and the general procedure described above compound **13a** was obtained as a white solid in 45% yield; m.p. 116–117 °C; silica gel TLC *R_f_ =* 0.24 (MeOH/DCM 10% *v*/*v*); IR (KBr, cm^−1^) 1669.8, 1670.7, 1605.8, 1558.4; δ_H_ (400 MHz, DMSO-*d*_6_) 1.37–1.41 (2H, m, 6′-H), 1.45–1.50 (4H, m, 5′-H), 2.33–2.38 (6H, m, 2′, 4′-H), 3.27 (2H, q, *J* = 6.4 Hz, 1′-H), 4.65 (2H, s, O*CH*_2_CO), 6.34 (1H, d, *J =* 9.6 Hz, 3-H), 7.00–7.04 (2H, m, 6, 8-H), 7.69 (1H, d, *J* = 8.4 Hz, 5-H), 8.03 (1H, d, *J* = 9.6 Hz, 4-H); δ_C_ (100 MHz, DMSO-*d*_6_) 24.9, 26.4, 36.9, 54.9, 58.3, 68.2, 102.7, 113.7, 113.9, 130.4, 145.1, 115.1, 161.1, 161.6, 167.7; *m*/*z* (ESI positive) (C_18_H_22_N_2_O_4_) 331.3 [M + H]^+^.


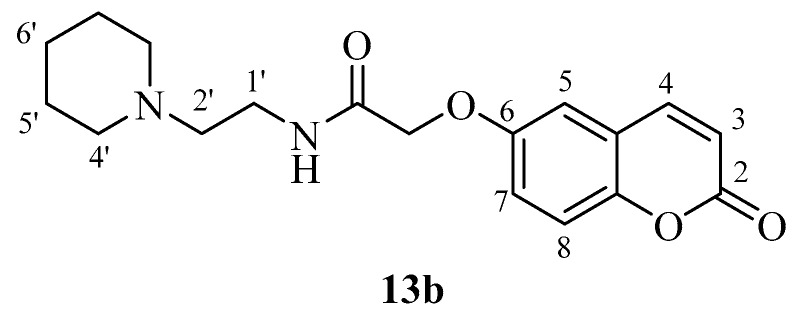


*2-((2-Oxo-2H-chromen-6-yl)oxy)-N-(2-(piperidin-1-yl)ethyl)acetamide* (**13b**)*.* Using **3b** and **13** as starting materials and the general procedure described above compound **13b** was obtained as a white solid in 45% yield; m.p. 115–116 °C; silica gel TLC *R_f_* = 0.30 (MeOH/DCM 10% *v*/*v*); IR (KBr, cm^−1^) 1701.6, 1670.4, 1610.0, 1554.8; δ_H_ (400 MHz, DMSO-*d*_6_) 1.38–1.39 (2H, m, 6′-H), 1.46–1.50 (4H, m, 5′-H), 2.35–2.42 (6H, m, 2′, 4′-H), 3.27 (2H, q, *J*= 6.6 Hz, 1′-H), 4.57 (2H, s, O*CH*_2_CO), 6.52 (1H, d, *J =* 9.6 Hz, 3-H), 7.29 (1H, dd, *J* = 2.9, 8.9 Hz, 7-H), 7.33 (1H, d, *J* = 2.9 Hz, 5-H), 7.41 (1H, d, *J =* 8.9 Hz), 7.96 (1H, t, *J* = 6.6 Hz, exchange with D_2_O, N*H*), 8.04 (1H, d, *J* = 9.6 Hz, 4-H); δ_C_ (100 MHz, DMSO-*d*_6_) 24.9, 26.4, 36.8, 54.8, 58.3, 68.4, 112.9, 117.6, 118.3, 120.1, 120.9, 144.9, 149.1, 154.9, 160.9, 168.0; *m*/*z* (ESI positive) (C_18_H_22_N_2_O_4_) 331.3 [M + H]^+^.

### 3.3. Soybean Lipoxygenase Inhibition Studies

A DMSO solution of the tested compound was incubated with sodium linoleate (0.1 mM) and 0.2 mL of soybean LOX solution (1/9 × 10^−4^ *w*/*v* in saline) in buffer pH 9 (tris) and at room temperature. The conversion of sodium linoleate to 13-hydroperoxylinoleic acid was recorded at 234 nm and compared with the standard inhibitor NDGA (IC_50_ = 0.45 μM). The results are given in Table 2 expressed as IC_50_ values or % inhibition at 100 μΜ [34].

### 3.4. CA Inhibition Assay

An Applied Photophysics (Oxford, UK) stopped-flow instrument has been used for assaying the CA catalysed CO_2_ hydration activity [36]. Phenol red (at a concentration of 0.2 mM) has been used as indicator, working at the absorbance maximum of 557 nm, with 20 mM HEPES (pH 7.5) as buffer, and 20 mM Na_2_SO_4_ (for maintaining constant the ionic strength), following the initial rates of the CA-catalyzed CO_2_ hydration reaction for a period of 10–100 s. The CO_2_ concentrations ranged from 1.7 to 17 mM for the determination of the kinetic parameters and inhibition constants. For each inhibitor at least six traces of the initial 5–10% of the reaction have been used for determining the initial velocity. The uncatalyzed rates were determined in the same manner and subtracted from the total observed rates. Stock solutions of inhibitor (0.1 mM) were prepared in distilled-deionized water and dilutions up to 0.01 nM were done thereafter with distilled-deionized water. Inhibitor and enzyme solutions were preincubated together for 6 h at 4 °C prior to assay, in order to allow for the formation of the E-I complex and for the active site mediated hydrolysis of the inhibitor [18,19]. Data reported in Table 1 show the inhibition after 6 h incubation, which led to the completion of the in situ hydrolysis of the coumarin and formation of the hydroxycinnamic acid [18,19]. The inhibition constants were obtained by non-linear least-squares methods using PRISM 3, as reported earlier [42,43,44,45,46,47,48,49,50] and represent the mean from at least three different determinations. The four CA isoforms were recombinant proteins obtained as reported earlier in our laboratory [42,43,44,45,46,47,48,49,50].

## 4. Conclusions

We report here a series of carboxamide derivatives of 6- and 7-substituted coumarins. They have been prepared by an original procedure starting from the corresponding 6- or 7-hydroxycoumarins which were alkylated with ethyl iodoacetate, then the obtained ester was converted to the corresponding carboxylic acid which was thereafter reacted with a series of aromatic/aliphatic/heterocyclic amines leading to the desired amides. The present study shows that these compounds represent a promising class of multi-targeting derivatives which can interact with several biological targets, in this case, lipoxygenase and carbonic anhydrases. Compounds **4a** and **4b** were potent LOX inhibitors, whereas many effective hCA IX inhibitors (K_I_s in the range of 30.2–30.5 nM) were detected in this study. Two compounds **4b** and **5b** showed the phenomenon of dual inhibition. Furthermore, these coumarins did not significantly inhibit the widespread cytosolic isoforms hCA I and II, whereas they were weak hCA IV inhibitors, making them hCA IX-selective inhibitors. As hCA IX and LOX are validated antitumor targets, these results are promising for the investigation of novel drug targets involved in tumorigenesis.

## Figures and Tables

**Table 1:** Inhibition data of CA I, II, IV and IX with compounds reported here and the standard sulfonamide inhibitor acetazolamide (**AAZ**) by a stopped flow CO_2_ hydrase assay [36].

Compound	K_I_ (nM) *			
	hCA I	hCA II	hCA IV	hCA IX
**2a**	>10,000	>10,000	5572	247.1
**2b**	>10,000	>10,000	>10,000	2044
**3a**	>10,000	>10,000	8480	290.2
**3b**	>10,000	>10,000	>10,000	194.9
**4a**	>10,000	>10,000	>10,000	165.7
**4b**	>10,000	>10,000	>10,000	30.5
**5a**	>10,000	>10,000	>10,000	83.7
**5b**	>10,000	>10,000	>10,000	30.2
**6a**	>10,000	>10,000	>10,000	2536
**6b**	>10,000	>10,000	>10,000	2785
**7a**	>10,000	>10,000	>10,000	200.6
**7b**	>10,000	>10,000	2649	201.9
**8a**	>10,000	>10,000	350.4	136.1
**8b**	>10,000	>10,000	848.3	145.6
**9a**	>10,000	>10,000	>10,000	2732
**9b**	>10,000	>10,000	>10,000	2041
**10a**	>10,000	>10,000	>10,000	2377
**10b**	>10,000	>10,000	>10,000	2147
**11a**	>10,000	>10,000	766.4	122.3
**11b**	>10,000	>10,000	>10,000	1962
**12a**	>10,000	>10,000	>10,000	>10,000
**12b**	>10,000	>10,000	>10,000	>10,000
**13a**	>10,000	>10,000	>10,000	1969
**13b**	>10,000	>10,000	>10,000	273.7
**AAZ**	250	12	74	25

* Mean from 3 different assays, by a stopped flow technique (errors were in the range of ±5−10% of the reported values, data not shown).

**Table 2:** In vitro inhibition of soybean LOX (IC_50_ μΜ or % LOX inhibition) [34] and lipophilicity values of 6- or 7- substituted coumarin derivatives **1**–**13** and their clogP values.

Compounds	ClogP ^a^	% LOX Inhibition at 100 Μμ ^b^	IC_50_ (μΜ) ^b^
**1a** (7-HC)	1.62	Reference compound	43 µM ^c^
**1b** (6-HC)	1.62	40%	nt ^e^
**2a ^d^**	1.89	16%	nt ^e^
**2b ^d^**	1.89	41%	nt ^e^
**3a ^d^**	1.03	50%	100 μΜ
**3b ^d^**	1.03	50%	100 μΜ
**4a**	2.60	96%	10 μΜ
**4b**	2.60	85%	10 μΜ
**5a**	2.39	45%	nt ^e^
**5b**	2.39	42%	nt ^e^
**6a**	2.53	63%	47 μΜ
**6b**	2.53	26%	nt ^e^
**7a**	2.36	50%	100 μΜ
**7b**	2.36	45%	nt ^e^
**8a**	2.76	43%	nt ^e^
**8b**	2.76	11%	nt ^e^
**9a**	2.80	33%	nt ^e^
**9b**	2.80	50%	100 μΜ
**10a**	1.11	79%	15 μΜ
**10b**	1.11	40%	nt ^e^
**11a**	0.89	77%	27 μΜ
**11b**	0.89	37%	nt ^e^
**12a**	0.91	56%	42.5 μΜ
**12b**	0.91	64%	16.5 μΜ
**13a**	2.32	50%	100 μΜ
**13b**	2.32	37.6%	nt ^e^
**NDGA**		93%	0.45 μM

Means within each column differ significantly (*p* < 0.05). ^a^ clogP values were measured by using the Biobyte C-QSAR [37]. ^b^ Values are means (±SD < 10%) of three or four different determinations. ^c^ From reference [38]. ^d^ Values are referred to **2a**, **3a**, **2b** and **3b** coumarin acetic acid derivatives [38]. ^e^ nt, not tested (IC_50_ values not found due to the fact that it may be >100 μΜ).
